# The critical role of threat detection and movement behavior assessment: Identifying key concepts, research scope, and gaps—a scoping review

**DOI:** 10.3389/fpsyg.2025.1627066

**Published:** 2025-10-01

**Authors:** Sandra Adiarte, Claus-Christian Carbon, Robin Orr, Elisa F. D. Canetti

**Affiliations:** ^1^Tactical Research Unit, Bond University, Gold Coast, QLD, Australia; ^2^Faculty of Health Sciences and Medicine, Bond University, Gold Coast, QLD, Australia; ^3^Department of General Psychology and Methodology, University of Bamberg, Bamberg, Germany

**Keywords:** concealed carry, detection, law enforcement, perception, non-verbal communication

## Abstract

Police officers face the challenging task of identifying potential threats daily. Failure to identify these threats soon enough can lead to physical injury, and, in the case of an armed offender, a fatal outcome. As such, the ability of an officer to identify an armed threat is critical. This scoping review aimed to identify and synthesize current literature regarding the ability of police officers or CCTV operators to identify an immediate source of threat. A systematic and repeated search of academic databases (Ovid Medline, PubMed, SportDiscus, Web of Science, and Embase) was finalized 26th of November 2024. All articles were screened for relevance against inclusion and exclusion criteria. A snowballing approach was employed, and the reference lists of pertinent studies were searched for additional sources of information. From an initial 14,812 records, four studies specifically met the eligibility criteria. All these studies evaluated the ability of law enforcement officers to identify threats and assessed the performance of the officers by investigating risk estimates and detection tests. Various video data-based tasks and tests have been used to assess the ability to identify upcoming or acute threats. Limited research has been published of studies investigating police officer concealed threat detection approaches, methods, and outcomes, highlighting a paucity of research in this critical field.

## Introduction

Police officers face various types of threats daily. These threats can include potentially aggressive and dangerous behaviors (Covington et al., 2014; Tiesman et al., 2018) and dealing with individuals suffering from psychological conditions (Yee et al., 2020). Whilst dealing with these individuals, officers and security personnel may need to detect and identify threats like concealed weapons (Pratihar and Yadav, 2014) or identify people, making them the target of their aggression (Schouten and Brennan, 2016; Johnson, 2019). Stop-and-search situations can go fatally wrong (Baumgartner et al., 2018) due to resistance from alleged offenders hoping to escape arrest and face associated charges, leading to around 10% of all police officers being assaulted and violently attacked each year (Bierie, 2017), with the number of assaults against officers rising (FBI, 2024). While the factors influencing these occurrences vary (Den Heyer, 2023) and the challenge of analyzing such incidents lies in the complexity of these encounters (Den Heyer, 2024), the ability to detect and identify concealed weapons and potential threats is of great relevance, as well as the necessity to gain an empirical understanding of threats via behavioral approaches (Sweet et al., 2017).

This ability to detect and identify potential threats sooner could allow police officers (and security personnel) to react more quickly or even prevent violent or malicious acts. However, this requires police officers to identify threats through observation and inherent contextualization of the situation in dynamic and versatile circumstances (Orr et al., 2020). The complexity of this requirement is espoused by research identifying physical assaults as the leading cause of injury in police officers (Tiesman et al., 2018). Thus, training officers to identify threats through observation as soon as possible is crucial when aiming to obtain safety at an officer level as without this training, severe consequences may ensue, especially if decisions are made incorrectly or are made too slowly. These consequences include personal, psychological, and professional impacts. Moreover, the decisions made may impact the safety of others, including their colleagues, the suspects, potential victims, as well as bystanders (Shortland et al., 2020).

Within the last decade, research in this field of threat observation has focused on psychophysiological markers of threat (Aikins et al., 2010; Marchak, 2013) and on developing machine learning algorithms to detect the concealment of objects, such as handguns from video images (Cohen et al., 2008; Darker et al., 2007), and concealed items via detection systems (Debnath and Bhowmik, 2021). Recent publications reviewed the methods applied in real-time early detection of weapons (Berardini et al., 2025) and describe the remaining challenges with these automated systems (Abi-Nader et al., 2025; Yadav et al., 2025). The consistency in their performance is challenged when it comes to detecting small objects, multiple weapons, in occlusion of weapons, and detecting weapons of different types fast and consistently in varying and complex settings (Berardini et al., 2025). Automated systems are ideally available 24/7 and, once established, seem to be a reliable support for surveillance tasks. Automated and deep learning systems have been successfully applied in areas like healthcare (Dias et al., 2020; Konstantinidis et al., 2021), sports (Tisserand et al., 2017), and education (Stefanidis et al., 2019). In security AI-based systems have outperformed other methods (Pisati et al., 2024). Ultimately, AI is based on the understanding of the relevant behaviors and movement patterns defined for detection and the design of the program it is attached to (Dimitropoulos et al., 2021). Some scientific projects have explored threat identification at a crowd level. However, detailed work on the specific movement cues observers use to recognize emergent threats within a crowd is absent (Brunye et al., 2014; Crundall and Eyre-Jackson, 2017). Other structured assessment tools such as the Behavioral Threat Assessment and Management (BTAM) program and the Terrorist Radicalization Assessment Protocol (TRAP-18) (Guldimann and Meloy, 2020; Nguyen et al., 2024)—employ evidence-based tools to assess individuals at risk of targeted violence or radicalization, respectively. Though effective, these tools rely on timely, multidisciplinary, collaborative data collection from various sources, which can often be fragmented, limiting their practical application when law enforcement officer is faced with imminent threat (Nguyen et al., 2024; Meloy et al., 2021; Amman and MacKizer, 2017). TRAP-18 has shown strong reliability and predictive validity through studies assessing lone-actor threats (Guldimann and Meloy, 2020; Challacombe and Lucas, 2019), but like BTAM, it demands comprehensive multidisciplinary data and time for accurate evaluations. However, while these approaches and assessment tools are reliable for assessing the chances of radicalization of lone-actor terrorists, they do not include offender-related, movement-based indicators of the threat focusing on the moments directly prior to the attack. While TRAP-18 assesses the risk of radicalization from a wealth of data, the evaluation and detailed analysis of the imminent threat are not very well investigated. In this regard, findings on behavioral or movement abnormalities should, in the best case, support the accurate assessment of the level of danger, and prevent impending attacks (Sweet et al., 2023). Having the skills to know how and what to observe could be advantageous for officers and security personnel when dealing with members of the public, especially given the versatility of tasks officers perform in various settings (Kleygrewe et al., 2022), any of which could include unexpected attacks.

This scoping review aimed to identify and summarize knowledge regarding police officers and security personnel’s observation skills as part of an imminent threat assessment involving a concealed weapon or item, or an impending criminal act. The secondary aims include: (a) offering insights into current best practices in relation to human observer detection of a threat of concealed weapons, (b) exploration of common themes and challenges related to this topic, (c) discussing core findings, and (d) identifying relevant gaps in the literature and suggest directions for further research.

## Methods

This scoping review followed the Preferred Reporting Items for Systematic reviews and Meta-Analyses extension for Scoping Reviews (PRISMA-SCR) guidelines (Page et al., 2021).

### Search strategy, information sources, and eligibility criteria

A list of relevant key search terms, derived from the concepts of “threat assessment,” “observation,” and “law enforcement,” was prepared by the lead author (SA) and confirmed by fellow authors and in consultation with Bond University Library Services. Supplementary material 1 provides search terms for PubMed, with complete search terms for all databases provided. As needed, an ‘*’ Boolean operator was used to ensure variants of the terms were captured. The keywords were trialled to ensure known articles in this field were captured. Prior to use, the search terms were contextualized to each of the selected databases using the systematic review accelerator (Clark et al., 2020). A search of five different databases known to publish research in this field (Ovid Medline, PubMed, SportDiscus, Web of Science, and Embase) was conducted on the 26th of November 2024.

To be included in this review studies had to: (a) investigate a threat involving (i.e., violent attack) and/or threat in the form of concealed carry or evaluate the detection of threat (e.g., impending criminal acts), (b) report on the levels of task performance of the relevant population (e.g., detection task, observational task, prediction task) systematically and experimentally, including observation of movement/ behavior in the form of schematic or realistic material (e.g., Closed Circuit Television (CCTV) footage), (c) involve law enforcement, military and/ or security personnel undergoing threat assessment/identification, and (d) be peer-reviewed original research published in English. Studies were excluded if they: (a) investigated other threats (i.e., medical conditions, diseases, prevention of political, social, personal, or environmental crisis), (b) did not involve experimental research and/or included qualitative data collection only, (c) used ‘still’ images or material that did not portray movement (i.e., photographs or inanimate objects or lack of motion or movement), (d) used an automated detection system instead of human-based assessment (i.e., real-time surveillance CCTV footage, automated detection technology), or (e) focused on after incident assessment of offender behavior and/or on historical, medical, or personal data for behavior assessment (i.e., structured assessment of historical data on violent attacks such as terrorist attacks).

### Screening and selection process

All records identified in the search were imported into a reference management software (EndNote, version 21, Clarivate Analytics, Philadelphia, United States), where duplicates were removed. After screening titles and abstracts, the full text of potentially relevant studies was obtained. Full-text studies were screened by the first author (SA), and findings were discussed with fellow authors (EFDC & RO) to minimize the risk of bias. The remaining relevant studies were printed, read, and processed in the following order: (1) Inspecting the title and keywords of the study, (2) reading and highlighting central information in the abstract, (3) cross-checking the keywords, the used terminology, and the investigated population (4) screening the conclusions section, the methods section and outcome measures, (5) identifying and marking common themes, approaches, methods and shared references (cross-referencing), and finally (6) reading the whole text highlighting relevant parts (if applicable) and extracting relevant information. The screening process focused on the mentioned inclusion/ exclusion criteria. The final decision on studies to be included was based on discussion and consensus between all authors.

### Data charting and items

Data charting included tabulating key information for structured documentation purposes. Due to the heterogeneity of methods, paradigms, and measures used in the different studies, data charting was reported in two steps: (1) a table giving a general overview, excluding the outcome measures, and (2) a table reporting the studies documenting the individual outcome measures. Items of interest included study characteristics (authors, country, participants), experimental methodology, type of threat (i.e., carrying a concealed firearm or unstable object, suicide bomb attack, impending criminal act), performance in threat assessment/identification task, and outcome measures (e.g., firearm detection tests, measurement of sensitivity to non-verbal cues or concealed firearm, risk estimates).

## Results

**Figure 1:**
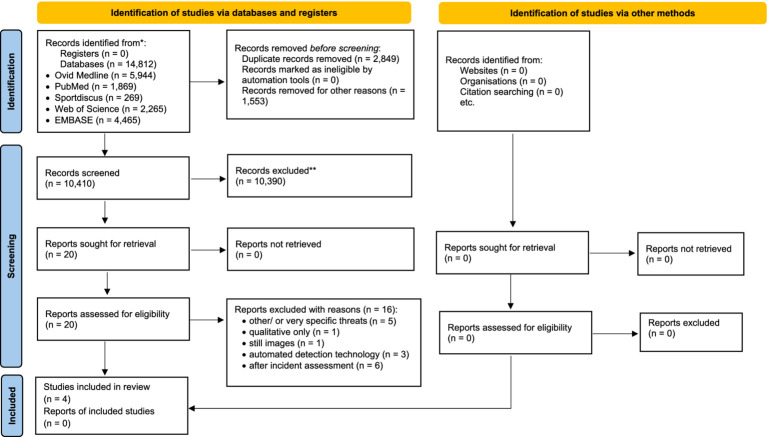
Prisma flow diagram.

The systematic search yielded 14,812 records. Duplicates (*n* = 2,849) were removed, and 1,553 records were excluded as not relevant to the research question (e.g., risk assessment in the context of injuries/ diseases/ therapy treatment). The titles and abstracts of the remaining 10,410 records were screened, and 20 full-text studies were assessed for eligibility. Fifteen studies were excluded, with reasons documented (Supplementary material 2), leaving four studies eligible to be included in the review (Blechko et al., 2008; Crundall and Eyre-Jackson, 2017; Sweet et al., 2023; Sweet et al., 2017) as outlined in Figure 1.

### Summary of included articles

Table 1 provides the characteristics of the four included studies. All included studies compared the ability of trained personnel (i.e., police officers, CCTV operators) and lay people (i.e., students, members of the public) to identify threats using video/motion analysis. However, given the differences in methodologies used, their findings will be reported separately.

**Table 1:** Characteristics of included studies.

Reference/year	Aim	Type of threat	Stimulus	Participants
Blechko et al. (2008)	Assess ability to detect a firearm, noting the emotional state of the surveillance target	Concealed firearm	Mock CCTV footage (12 actors individually filmed)	Total n = 16 n = 8 CCTV operators (*M*_age_ = 37; *SD* = 10 yrs.; *M*_exp_ = 5; *SD* = 3 yrs) n = 8 lay people (*M*_age_ = 47; *SD* = 12 yrs)
Crundall and Eyre-Jackson (2017)	Assess the ability to predict criminal incidents based on non-verbal behavior	Impending criminal act	Real CCTV footage (10 short clips)	Total n = 60 n = 30 LEOs (*M*_age_ = 36; *SD* = 9, range 21–56 yrs.; *M*_exp_ = 10; SD = 7 yrs) n = 30 lay people (*M*_age_ = 36; *SD* = 12, range 20–63 yrs)
Sweet et al. (2017)	Assess the ability detect concealment of a weapon or device	Concealment of weapon or unstable object	*Experiment 1*: mock video data (1 male target, 1 camera angle)	*Experiment 1:* Total n = 107 n = 51 LEOs (*M*_age_ = 41; *SD* = 9, range 27–64 yrs.; *M*_exp_ = 15; *SD* = 9 yrs) n = 56 students (*M*_age_ = 19.; *SD =* 1, range 18–24 yrs)
			*Experiment 2*: mock video data (3 male targets, 2 camera angles)	*Experiment 2:* Total n = 104 n = 49 LEOs (*M*_age_ = 41; *SD =* 9, range 28–64 yrs.; *M*_exp_ = 15; *SD* = 9 yrs) n = 55 students (*M*_age_ = 19; *SD =* 1, range 18–24 yrs)
			*Experiment 3*: mock video data (2 male targets, 4 camera angles)	*Experiment 3:* Total n = 104 n = 55 LEOs (*M*_age_ = 40; *SD =* 9, range 25–64 yrs.; *M*_exp_ = 14; *SD* = 8 yrs) n = 49 students (*M*_age_ = 20; *SD =* 2, range 18–26 yrs)
Sweet et al. (2023)	Assess the ability to detect an innocuous object or device	Detection of an innocuous object or unstable device	*Experiment 1*: mock video data (1 male or female target, 2 camera angles)	*Experiment 1:* Total n = 120 n = 50 LEOs (*M*_age_ = 38; *SD* = 8, range 24–62 yrs.; *M*_exp_ = 12; *SD* = 10 yrs) n = 70 students (*M*_age_ = 29; *SD* = 3, range 18–43 yrs)
				*Experiment 3: see* Sweet et al. (2017)

LEOs, law enforcement officers; CCTV, Closed Circuit Television; M, mean; M_exp_, mean experience (in years); SD, standard deviation; yrs, years.

Sweet et al. (2017) systematically investigated the ability of officers (age = 40.53 ± 8.83 years; years of experience = 14.53 ± 9.40) and naïve control group participants (students ranging from 18 to 24 (19.12 ± 1.35) years of age) to detect concealed weapons in schematic video footage in three discriminative studies. In their first experiment, officers and naïve controls performed significantly better than chance in discriminating threat (Cohen’s *d* = 0.27 95% CI [0.08, 0.46], *t* (106) = 2.79, *p* < 0.001) by identifying a concealed handgun via a classic signal detection paradigm. In their second experiment, participants had to assess whether one of several individuals carrying a backpack was carrying a concealed unstable device. Both investigated groups performed better than chance (*d* = 0.47 95% CI [0.31, 0.63], *t* (103) = 5.74, *p* < 0.001). In their final experiment, participants were asked to choose between two individuals, deciding which one of the two was concealing an unstable device in their backpack. In this study, police officers and the naïve control group performed at chance level (*p_c_* = 0.50 95% CI [0.47, 0.53], *t* (103) = 0.11, *p* = 0.91). All three experiments included customized schematic video footage of different scenarios and lengths. The researchers varied the threat stimuli (i.e., firearm, improvised explosive device (IED)) and choice paradigms (classic signal paradigm, compound decision paradigm, two-alternative forced choice paradigm). While officers and naïve controls performed significantly better than chance in the first experiment, outcomes of the second and third experiment showed no significant differences between laypeople and police officers in identifying weapon concealment or threat detection in form of an IED (Sweet et al., 2017).

In a later series of studies, Sweet et al., compared the perception of patterns of movement and the detection of concealment in law enforcement officers and naïve observers with a different team of colleagues. They conducted a series of experiments using a Lens Model framework (Sweet et al., 2023). The goal of the first study was to investigate the ability of law enforcement officers to identify and articulate behavioral cues in videos indicating a person is carrying a concealed weapon or item. Participants (*n* = 120) were recruited for the first study. These participants included 50 law enforcement officers (25 females) and 70 students (30 females). Officers ranged in age from 24 to 62 (age = 38.00 ± 7.60 years) and had a mean of 12.38 (±9.68) years of experience. Student participants ranged in age from 18 to 43 years (age = 29.15 ± 3.25). All participants in this experiment showed poor ability to distinguish between individuals concealing a handkerchief and those who were not (Sweet et al., 2023), with officers being less accurate than laypersons (*M* = 0.19 and 0.51, *SD* = 0.94 and 1.05, respectively).

The second study used material from the experiment of (Sweet et al., 2017). Fifty-one law enforcement officers (female = 6) and 56 students (male = 37) participated. The officers ranged in age from 27 to 64 years (age = 40.53 ± 8.83 years) and had a mean of 14.53 (± 9.40) years of experience. Student participants ranged in age from 18–24 years (age = 19.12 ± 1.35 years).

Participants had difficulties in identifying specific diagnostic behaviors for concealment and furthermore struggled in detecting whether the concealed object was a firearm, an unstable device, or a handkerchief. In summary, their second study suggests that discriminating between individuals who are concealing and those who are not can hardly be obtained by focussing on a few reliable behavioral cues or movement patterns. The outcome revealed poor judgment capabilities for law enforcement and naïve observers. Recommendations include further research to enable empirically supported training for the accurate and appropriate articulation of relevant behaviors, allowing for the clear justification when searching individuals who carry concealed weapons (Sweet et al., 2023).

Blechko et al. (2008) conducted an experiment comparing CCTV operators’ and laypeople’s skills in detecting concealed carry of individuals (a firearm or an innocuous object / a bottle) by viewing CCTV footage. The assessments were conducted in two steps: (1) a profile of the nonverbal sensitivity test (PONS Test) as designed by Rosenthal and colleagues measured the participant’s ability to read body language (decoding silent, non-verbal behavioral cues), and (2) a firearm detection test investigating the participants’ skill in detecting handgun carrying by using modified CCTV footage (Rosenthal et al., 1979). The outcome of the PONS test analyzing overall movement was similar between laypersons and CCTV operators (0.72 *±* 0.06 and 0.71 *±* 0.06, respectively). Similarly, the sensitivity to detect concealed firearms was not significantly different between laypersons and CCTV operators (*U* = 16.00; *r =* −0.42; *p* = 0.105). Notably, this study identified a significant negative correlation between the emotional state of the observed individuals, particularly when demonstrating sensation-seeking behavior, and an observer’s sensitivity to detect concealment (*r_s_* = −0.60; *n* = 12; *p* = *0*.040). Although their findings demonstrated average success in the detection task, their outcomes showed that the emotions experienced by the individual carrying a concealed firearm could support the detection of concealed carry (Blechko et al., 2008).

Crundall and Eyre-Jackson (2017) investigated if experienced police officers had a superior ability to detect impending criminal acts when compared to the general public. The researchers compared police officers (age = 36 *±* 9.0 years, range 21–56 years, *M* = 10 years in service) and members of the general public (age = 35.76 *±* 12.0 years, range 20–63 years) in their accuracy for discriminating criminal intent by watching CCTV footage. Participants were randomly assigned to one of the two tested video display conditions (freeze frame or occlusion). In addition, the data also included control clips containing no criminal actions. Overall, participants responded significantly slower to the control clips than the crime clips [*F*(1,54) = 12.81*, MSe* = 1.24 *p <* 0.05, control clips mean = 3.07 s; crime clips mean = 2.33 s]. A main effect regarding the participant group was found [*F*(1,54) = 4.2*, MSe* = 125.4*, p* < 0.05], with police officers performing significantly better than members of the general public (55.7% vs. 49.6%) and the officers having the fastest response time in correctly identifying crime clips [*F*(1,54) = 3.94*, MSe =* 1.24, *p =* 0.05]. Given this experimental setup (with clips being obscured prior to the criminal act), the researchers’ findings support those officers attended key components of the given scenes in an appropriate and timely way. Officers were significantly better and faster in the detection task than members of the public (Crundall and Eyre-Jackson, 2017).

In summary, the four presented studies (Blechko et al., 2008; Crundall and Eyre-Jackson, 2017; Sweet et al., 2023; Sweet et al., 2017) investigated the ability of trained personnel (police officers and CCTV operators) to detect threats in the form of concealed firearms or objects alongside control groups of students and members of the general public. A common finding in the four studies was that most participants performed at chance level with trained personnel not performing much better than laypeople in detecting concealed objects or threats (Blechko et al., 2008; Sweet et al., 2017; Sweet et al., 2023). In only one study police officers outperformed lay people significantly (Crundall and Eyre-Jackson, 2017).

Although all studies used videos as stimuli, it must be noted that the four included studies used a variety of paradigms, materials, and methods in their investigations which impede comparability. The volume of evidence from these studies suggests that there is a major need for original and interventional research in this field.

## Discussion

This scoping review aimed to identify literature and summarize knowledge about threat identification by human observers, explore common themes and challenges in this topic, and identify gaps in the literature to guide future research in threat identification. The current research focus, identified in this review, was on threat identification based on video material (i.e., CCTV footage) rather than investigating the human skill of acute *ad hoc* threat detection. The terminology and methodologies used across the studies were inconsistent, making comprehensive comparisons, volume of evidence summation, and direct transfer of outcomes to practice problematic.

Little systematic research was identified in this review that was specifically conducted on analyzing the performance of human detection tasks when aiming to identify threats. While threat assessment is typically conducted by trained experts in clinical settings prior to or after a critical incident (Meloy et al., 2012). Underpinned by the fact that human observers have been shown to be good at recognizing biological movement patterns (Johansson, 1973), the assessment of an imminent threat must be done in the field to provide ecologically valid results. Furthermore, performing well under extreme pressure is an essential requirement for law enforcement officers (Heusler and Sutter, 2020). Thus, officers must be able to recognize critical visual information (Fallon et al., 2014) and assess and react immediately according to an emerging situation (Murray et al., 2024). Vickers and Lewinski (2012) demonstrated that the ability to control gaze and focus of attention under threat result in better shooting accuracy, decision making, and firing speed. While this demonstrates that gaze control and focus of attention under threat are crucial, mechanisms behind how these are maintained during life-threatening situations still needs to be elucidated. Law enforcement officers have been victims of targeted violence, which makes a re-evaluation of traditional practices necessary (Harris and Lurigio, 2012). While some research does suggest that there is a difference in the nonverbal behavior of non-offenders and offenders (Koller et al., 2015), whereby criminal investigators were able to recognize thieves prior to them committing a crime, the scientific examination of the movement patterns and behaviors of attackers that occur immediately before, during, or after an attack is lacking (Dias et al., 2020; Konstantinidis et al., 2021; Tisserand et al., 2017; Stefanidis et al., 2019; Dimitropoulos et al., 2021; Brunye et al., 2014). The nature of policing duties yields a call for evidence-based, objective, and describable behavior patterns to train and support law enforcement officers in using a more diagnostic approach to detect threats, like the concealment of objects or weapons (Sweet et al., 2023). However, the volume of evidence to inform such training for police officers (and security officers) is limited.

The observation and analysis of behavior is a complex area to investigate, with numerous factors to consider and as such, it is an area that should not be neglected in favor of Artificial Intelligence (AI). As such, further research investigating and formalizing an approach to identify movement patterns and behaviors of attackers that occur immediately before, during, or after an attack is needed. Once these factors have been established and validated, officers and AI programs can be trained using a systematic and evidence-based approach.

### Limitations

A limitation to this review was the scarcity of finding sufficient peer-reviewed, published research studies investigating this field to provide “best practice guidelines” for this framework to train threat assessment. The diversity of frameworks, forms of tasks and methodologies applied inhibits direct comparability of research outcomes. While critical for establishing evidence-based best practice, the inability to review and cite grey literature of classified documents (anticipated given this field of research, where security and safety issues are inevitably involved), limits broader knowledge development. A second-order impact of this security and safety limitation means that work may not be shared across agencies, thus limiting the cohesive development of an evidence-based system through which to inform training, be it for officers or AI programs.

## Conclusion

Aiming to obtain the ‘street-level’ safety of an officer, training to identify threats as soon as possible is crucial when wanting to mitigate an immediate threat. Findings from the four studies informing this scoping review, include a diversity of approaches and outcomes, demonstrate a clear shortage of research in this area through which to build a volume of evidence to support best practice training. Research into this gap is necessary to assist in the prevention of life-threatening attacks on officers and larger-scale attacks on the community by individuals, as well as create more safety for presumable offenders. A focus on scenario-based experimental settings and detection tasks on real-life video footage could produce more transferable outcomes. To enhance future training and elevate the chances for safety for all involved, understanding the behaviors and movement patterns occurring prior to an attack needs to be at the core of future research interests. Interdisciplinary research projects should aim to answer questions like: What are the key movement patterns visible when carrying concealed? How can these patterns be described and explained? Consistency in methodology and research approach across a larger series of studies would support reliable and valid outcomes and enable the comparison of findings across the field. Structured basic research would furthermore facilitate the application of findings into AI-based systems. Stakeholders, scholars and practitioners aiming to understand the relevant behaviors related to immediate threats would benefit from this type of future research.

## Data Availability

The raw data supporting the conclusions of this article will be made available by the authors, without undue reservation, if requested.
