# Supplementary material for: The critical role of threat detection and movement behavior assessment: Identifying key concepts, research scope, and gaps—a scoping review
Source: Front Psychol. 2025 Oct 1;16:1627066. doi: 10.3389/fpsyg.2025.1627066 (PMC12520921; doi:10.3389/fpsyg.2025.1627066)
Supplement: Supplementary file 1 [file Supplementary_file_1.docx]

Supplementary Material 1- Search terms per database

| **DATABASE** | **SEARCH TERMS** |
| --- | --- |
| Ovid Medline | Military Science OR "military science" OR "military sciences“ OR (science and military) OR military OR Military Personnel OR "military personnel“ OR "air force personnel“ OR "army personnel“ OR submariner OR submariners OR marines OR "navy personnel“ OR sailor OR sailors OR "coast guard“ OR soldier OR soldiers OR tactical OR Law Enforcement OR "law enforcement“ OR SWAT OR "special weapons and tactics“ OR Police OR police OR police* OR cop OR cops OR sheriff OR army OR "armed forces“ OR "service members“ OR (service and members) OR officer OR officers **AND** Movement OR movement OR Behavior OR Observation OR ((behaviour* or behavior*) and (analysis or observation)) OR LMA OR "Laban Bartenieff Movement Studies“ OR Laban movement OR Rudolf Laban OR (Kinesics or Bernese or Labanotation or Benesh Movement Notation) OR "movement coding system“ OR "movement coding“ OR Gait Analysis **AND** risk OR threat OR Risk Assessment Risk OR "threat assessment"OR Weapons OR weapon OR inciden* OR gun OR guns OR firearm OR firearms OR Firearms OR (conceal or conceals or concealed or concealment or cover or covered or hide or hidden) OR "concealed weapon detection" OR danger* |
| PubMed | (((((((((((("Military Science"[Mesh]) OR Tactical) OR "Law Enforcement"[Mesh]) OR "law enforcement") OR "military science") OR SWAT) OR “Tactical Response”) OR "Police"[Mesh]) OR police) OR sheriff)) AND (((((((("Movement"[Mesh] AND "Observation"[Mesh]))) OR ((movement AND observation))) OR ((Movement) AND (Observation OR analysis OR system OR quality OR characteristics OR training OR “Education"[Mesh]))) OR (("Behavior"[Mesh] **AND** "Observation"[Mesh]))) OR "Behavior/analysis"[Mesh]) OR (((behaviour* OR behavior*)) AND (analysis OR observation)))) **AND** (((((((((("Risk"[Mesh]) OR risk) OR threat) OR "Risk Assessment"[Mesh]) OR “Risk assessment”) OR “Threat assessment”) OR "Weapons"[Mesh]) OR weapon*) OR danger*) OR inciden*) |
| SportDiscus | (("military science" OR "military sciences" OR military OR "military personnel" OR "air force personnel" OR "army personnel" OR submariner OR submariners OR marines OR marine OR "navy personnel" OR sailor OR sailors OR "coast guard" OR soldier OR soldiers OR tactical OR "law enforcement" OR SWAT OR "special weapons and tactics" OR police* OR cop OR cops OR sheriff OR army OR "armed forces" OR "service members" OR officer OR officers) ) **AND** (((Movement AND  (behavior OR behaviour OR analysis OR observation))) OR LMA OR "Laban movement" OR "Rudolf Laban" OR Kinesics OR Bernese OR Labanotation OR "Benesh Movement Notation" OR "Gait Analysis") **AND** (Risk OR threat OR weapon OR weapons OR inciden* OR gun OR guns OR firearm OR firearms OR conceal OR conceals OR concealed OR concealment OR cover OR covered OR hide OR hidden OR danger*) |
| Web of Science | ("military science" OR "military sciences" OR military OR "military personnel" OR "air force personnel" OR "army personnel" OR submariner OR submariners OR marines OR marine OR "navy personnel" OR sailor OR sailors OR "coast guard" OR soldier OR soldiers OR tactical OR "law enforcement" OR SWAT OR "special weapons and tactics" OR Police* OR cop OR cops OR sheriff OR army OR "armed forces" OR "service members" OR officer OR officers) **AND** ((Movement AND (behavior OR behaviour OR analysis OR observation))) OR LMA OR "Laban movement" OR "Rudolf Laban" OR Kinesics OR Bernese OR Labanotation OR "Benesh Movement Notation" OR "Gait Analysis“) **AND** (Risk OR threat OR weapon OR weapons OR inciden* OR gun OR guns OR firearms OR firearms OR (conceal OR conceals OR concealed OR concealment OR cover OR covered OR hide OR hidden) OR danger*) |
| Embase | ('Military Science'/exp) OR Tactical OR 'Law Enforcement'/exp OR "law enforcement" OR "military science" OR SWAT OR "Tactical Response" OR 'Police'/exp OR police OR sheriff **AND** (((((((('Movement'/exp AND 'Observation'/exp))) OR ((movement AND observation ))) OR ((Movement ) AND (Observation OR analysis OR system OR quality OR characteristics OR training OR 'Education'/exp))) OR (('Behavior'/exp AND 'Observation'/exp))) OR 'Behavior/analysis'/exp) OR (((behaviour* OR behavior* )) AND (analysis OR observation ))) **AND** (((((((((('Risk'/exp) OR risk ) OR threat ) OR 'Risk Assessment'/exp) OR "Risk assessment" ) OR "Threat assessment" ) OR 'Weapons'/exp) OR weapon* ) OR danger* ) OR inciden* ) |

Supplementary Material 2: Excluded Articles with Reasons

| **Reason for Exclusion** | **Reference** |
| --- | --- |
| Other/ or very specific threats (n=4) | [1-4] |
| Qualitative only (n=1) | [5] |
| Still images (n=1) | [6] |
| Automated detection technology (n=3) | [7-9] |
| After incident assessment (n=6) | [10-15] |

References:

1. Bloom, A.H., Whack-A-Mole Reasonable Suspicion. Cal. L. Rev., 2024. **112**: p. 1129.
2. Denault, V., et al., The analysis of nonverbal communication: the dangers of pseudoscience in security and justice contexts. *Anuario de Psicología Jurídica*, 2020.
3. Faccini, L.A., C. S., The role of personal identity in acts of targeted violence: An important factor in risk and threat assessment. *International Journal on Disability and Human Development* 2022. **21**(4): p. 415
4. Horn, R., et al., Assessing between-officer variability in responses to a live-acted deadly force encounter as a window to the effectiveness of training and experience. *Ergonomics*, 2024. **67**(8): p. 1035-1050.
5. Sieben, A., J. Schumann, and A. Seyfried, Collective phenomena in crowds—Where pedestrian dynamics need social psychology. *PLoS one*, 2017. **12**(6): p. e0177328.
6. Jiang, X., et al., MAGNet: A camouflaged object detection network simulating the observation effect of a magnifier. Entropy, 2022. **24**(12): p. 1804.
7. Bhatt, A. and A. Ganatra, Weapon operating pose detection and suspicious human activity classification using skeleton graphs. *Mathematical Bio-sciences and Engineering*, 2023. **20**(2): p. 2669-2690.
8. Chen, H.-M., et al., Imaging for concealed weapon detection: a tutorial overview of development in imaging sensors and processing. *IEEE signal processing Magazine*, 2005. **22**(2): p. 52-61.
9. Murray, N.P., et al., The eyes have it! Functional field of view differences between visual search behavior and body-worn camera during a use of force response in active-duty police officers. *Police Practice and Research*, 2024. **25**(4): p. 490-497.
10. Goodwill, A. and J.R. Meloy, Visualizing the relationship among indicators for lone actor terrorist attacks: Multidimensional scaling and the TRAP-18. *Behav Sci Law*, 2019. **37**(5): p. 522-539.
11. Kantor, M.A., et al., The 21-foot principle: Effects of age and sex on knife attack characteristics. Journal of forensic and legal medicine, 2024. **101**: p. 102637.
12. Meloy, J.R., The Operational Development and Empirical Testing of the Terrorist Radicalization Assessment Protocol (TRAP-18). *J Pers Assess*, 2018. **100**(5): p. 483-492.
13. Meloy, J.R. and J. Genzman, The clinical threat assessment of the lone-actor terrorist. *Psychiatric Clinics of North America*, 2016. **39**(4): p. 649-662.
14. Meloy, J.R., et al., The Concept of Identification in Threat Assessment. *Behavioral Sciences & the Law*, 2015. **33**(2-3): p. 213-37.
15. Meloy, J.R., et al., The role of warning behaviors in threat assessment: An exploration and suggested typology. Behavioral sciences & the law, 2012. **30**(3): p. 256-279.
