# Supplementary material for: The critical role of threat detection and movement behavior assessment: Identifying key concepts, research scope, and gaps—a scoping review
Source: Front Psychol. 2025 Oct 1;16:1627066. doi: 10.3389/fpsyg.2025.1627066 (PMC12520921; doi:10.3389/fpsyg.2025.1627066)
Supplement: Supplementary file 2 [file Supplementary_file_2.docx]

Supplementary Material 2 - Excluded Articles with Reasons

| **Reason for Exclusion** | **Reference** |
| --- | --- |
| Other/ or very specific threats (n=4) | [1-4] |
| Qualitative only (n=1) | [5] |
| Still images (n=1) | [6] |
| Automated detection technology (n=3) | [7-9] |
| After incident assessment (n=6) | [10-15] |

References:

1. Bloom, A.H., Whack-A-Mole Reasonable Suspicion. Cal. L. Rev., 2024. **112**: p. 1129.
2. Denault, V., et al., The analysis of nonverbal communication: the dangers of pseudoscience in security and justice contexts. *Anuario de Psicología Jurídica*, 2020.
3. Faccini, L.A., C. S., The role of personal identity in acts of targeted violence: An important factor in risk and threat assessment. *International Journal on Disability and Human Development* 2022. **21**(4): p. 415
4. Horn, R., et al., Assessing between-officer variability in responses to a live-acted deadly force encounter as a window to the effectiveness of training and experience. *Ergonomics*, 2024. **67**(8): p. 1035-1050.
5. Sieben, A., J. Schumann, and A. Seyfried, Collective phenomena in crowds—Where pedestrian dynamics need social psychology. *PLoS one*, 2017. **12**(6): p. e0177328.
6. Jiang, X., et al., MAGNet: A camouflaged object detection network simulating the observation effect of a magnifier. Entropy, 2022. **24**(12): p. 1804.
7. Bhatt, A. and A. Ganatra, Weapon operating pose detection and suspicious human activity classification using skeleton graphs. *Mathematical Bio-sciences and Engineering*, 2023. **20**(2): p. 2669-2690.
8. Chen, H.-M., et al., Imaging for concealed weapon detection: a tutorial overview of development in imaging sensors and processing. *IEEE signal processing Magazine*, 2005. **22**(2): p. 52-61.
9. Murray, N.P., et al., The eyes have it! Functional field of view differences between visual search behavior and body-worn camera during a use of force response in active-duty police officers. *Police Practice and Research*, 2024. **25**(4): p. 490-497.
10. Goodwill, A. and J.R. Meloy, Visualizing the relationship among indicators for lone actor terrorist attacks: Multidimensional scaling and the TRAP-18. *Behav Sci Law*, 2019. **37**(5): p. 522-539.
11. Kantor, M.A., et al., The 21-foot principle: Effects of age and sex on knife attack characteristics. Journal of forensic and legal medicine, 2024. **101**: p. 102637.
12. Meloy, J.R., The Operational Development and Empirical Testing of the Terrorist Radicalization Assessment Protocol (TRAP-18). *J Pers Assess*, 2018. **100**(5): p. 483-492.
13. Meloy, J.R. and J. Genzman, The clinical threat assessment of the lone-actor terrorist. *Psychiatric Clinics of North America*, 2016. **39**(4): p. 649-662.
14. Meloy, J.R., et al., The Concept of Identification in Threat Assessment. *Behavioral Sciences & the Law*, 2015. **33**(2-3): p. 213-37.
15. Meloy, J.R., et al., The role of warning behaviors in threat assessment: An exploration and suggested typology. Behavioral sciences & the law, 2012. **30**(3): p. 256-279.
